# Regional hypothalamic resting state connectivity with limbic structures: An ultra-high field functional magnetic resonance imaging investigation

**DOI:** 10.1162/IMAG.a.46

**Published:** 2025-06-16

**Authors:** Rebecca V. Robertson, Noemi Meylakh, Lewis S. Crawford, Leana Sattarov, Fernando A. Tinoco Mendoza, Paul M. Macey, Vaughan G. Macefield, Kevin A. Keay, Luke A. Henderson

**Affiliations:** School of Medical Sciences (Neuroscience), Brain and Mind Centre, University of Sydney, Sydney, Australia; UCLA School of Nursing and Brain Research Institute, University of California, Los Angeles, CA, United States; Department of Neuroscience, Monash University, Melbourne, VIC, Australia

**Keywords:** hypothalamus, resting-state connectivity, prefrontal, amygdala, bed nucleus stria terminalis, thalamus

## Abstract

The hypothalamus contains discrete nuclei, each with distinct functions. Although human studies have investigated hypothalamo-cortical interactions in disease states, due to spatial resolution limitations, these studies were not able to explore interactions of specific hypothalamic subnuclei. Here, we used ultra-high field (7-Tesla) functional magnetic resonance imaging to explore resting connectivity patterns in six discrete hypothalamic regions in awake humans (n = 150). We found that the lateral, posterior, and paraventricular hypothalamus demonstrated widespread connectivity to all prefrontal subregions (dorsolateral prefrontal [dlPFC], inferior frontal, orbital and polar frontal [OPF], medial prefrontal [mPFC], cingulate cortices) as well as the insula, and that it was the lateral hypothalamus which displayed the most widespread connectivity. The anterior hypothalamus demonstrated connectivity with the dlPFC, OPF, mPFC and insula, while the dorsomedial hypothalamus showed connectivity to the dlPFC, OPF, mPFC, and insula. In contrast, the ventromedial hypothalamus showed no significant connectivity to either prefrontal or insula regions. All hypothalamic regions showed connectivity with the bed nucleus of the stria terminalis, and all except for the anterior nucleus showed connectivity with numerous amygdala subregions. The ventromedial and, to a lesser extent, the posterior hypothalamus showed connectivity with the medial dorsal, ventral anterior, and ventrolateral thalamus. This study shows that the hypothalamus, the PFC, the insula, and associated limbic areas strongly interact, but these interactions are not equal throughout both the hypothalamus and associated regions. These findings are broadly consistent with preclinical neuroanatomical tract tracing data, but they provide new insights into potential differences in connectivity patterns in humans.

**Table:** 

*List of abbreviations for hypothalamic regions used as seeds*			
AHA		anterior hypothalamic area	
DMH		dorsomedial hypothalamus	
LHA		lateral hypothalamic area	
PHA		posterior hypothalamic area	
PVH		paraventricular hypothalamus	
VMH		ventromedial hypothalamus	

*List of abbreviations for regions used as targets*			
24pr	24 prime	LP	lateral posterior
33pr	33 prime	L-Sg	limitans suprageniculate
47l	47 lateral	MD	mediodorsomedial
8BL	8B lateral	MeA	medial amygdala
9m	9 middle	MGN	medial geniculate
9a	9 anterior	MI	middle insula
9p	9 posterior	MV(Re)	reuniens
a10p	anterior 10p	OFC	orbital frontal complex
a32pr	32 prime	OPF	orbital and polar frontal
a47r	anterior 47r	p10p	posterior 10p
a9-46v	anterior 9-46v	p24	posterior 24
AAIC	anterior agranular insular	p24pr	posterior 24 prime
ACo	anterior cortical amygdaloid	p32pr	p32 prime
AHi	amygdalohippocampal area	p47r	posterior 47r
AHA	anterior hypothalamic area	Pf	parafascicular
AV	anteroventral thalamus nucleus	PI	para-insula
AVI	anterior ventral insular area	pOFC	posterior orbital frontal complex
BL	basolateral	Pol1	posterior insula 1
BLVM	basolateral ventromedial	Pol2	posterior insula 2
BM	basomedial	PuA	pulvinar anterior
BNST	bed nucleus stria terminalis	Pul	pulvinar inferior
Ce	central	PuL	pulvinar lateral
CeM	central medial	PuM	pulvinar medial
CL	central lateral	RSC	retrosplenial cortex
CM	centralmedian	VA	ventral anterior
d32	dorsal 32	VLa	ventral lateral anterior
Ig	Insular granular complex	VLp	ventral lateral posterior
La	lateral	VLA	ventral lateral anterior
LD	laterodorsal	VPL	ventral posterolateral
LGN	lateral geniculate		

## 1 Introduction

The hypothalamus is a key homeostatic regulatory region whose subregions mediate a wide range of functions, including the regulation of body temperature, hunger and thirst, sleep, as well as emotional expression and pain. Each of these functions is mediated by distinct subnuclei that preclinical studies have shown to possess different afferent and efferent projections (Williams et al., 2001). While the circuitry underpinning many hypothalamic functions have been defined in preclinical studies (Abrahamson & Moore, 2001;Bonnavion et al., 2016;Henderson & Macefield, 2021;Qin et al., 2018), studies defining projections to and from specific hypothalamic subnuclei to brain regions involved in processing higher order functions such as emotional processing are lacking for humans.

Recent studies have explored the role of the hypothalamus in emotional processing, highlighting its strong connectivity with the limbic system and detailing impaired functional connectivity, that is, functional magnetic resonance imaging (fMRI) signal intensity covariation between brain regions, in conditions categorised by altered emotional processing (Kong et al., 2021;Tu et al., 2018;Wang et al., 2019;Xiao et al., 2024). However, these studies lacked the ability to investigate the specific contributions of discrete hypothalamic nuclei and their unique connectivity patterns. Non-human tract tracing studies have shown that the hypothalamus is closely associated with prefrontal (PFC) brain regions, and that distinct hypothalamic nuclei are connected to different prefrontal regions as well as other limbic structures (Floyd et al., 2001;Freedman et al., 2000;Öngür et al., 1998). These tracing studies show clearly that different hypothalamic nuclei project to different parts of the limbic system and therefore mediate different functions. Given these differential projection patterns, it is therefore important to begin to define the connectivity patterns of key hypothalamic nuclei to provide a platform to explore these connections in disease state in humans.

Although it is almost impossible to explore the patterns of anatomical connectivity of discrete hypothalamic nuclei in living humans, it is possible to determine resting connectivity patterns between the hypothalamus and other brain regions. Resting-state connectivity analysis essentially explores signal covariations between brain regions and while fMRI signal intensity covariations can reflect direct anatomical neural connections, two brain regions that are not directly anatomically connected can also display robust signal covariation. Resting-state connectivity using fMRI has been applied to investigate the hypothalamus in a range of disease states with significant emotional components, including depression (Sudheimer et al., 2015;Wang et al., 2019), migraine (Meylakh et al., 2020), and chronic pain conditions (Kong et al., 2021). However, these studies were not able to explore connectivity patterns from discrete hypothalamic nuclei largely due to limitations of available MRI scanners.

The advent of ultra-high field human MRI scanners now allows for millimetre spatial resolution and thus the exploration of connectivity patterns of discrete hypothalamic nuclei. Given this, the aim of this study is to use ultra-high field 7-Tesla fMRI to explore hypothalamic nuclei connectivity with the prefrontal cortex and associated limbic areas, at rest in awake humans. We propose that the resulting connectivity patterns will provide a platform for future studies exploring hypothalamic connectivity strength changes in disease states, and particularly those characterised by changes in emotional regulation.

## 2 Methods

### 2.1 Participants

**Fig. 1.**
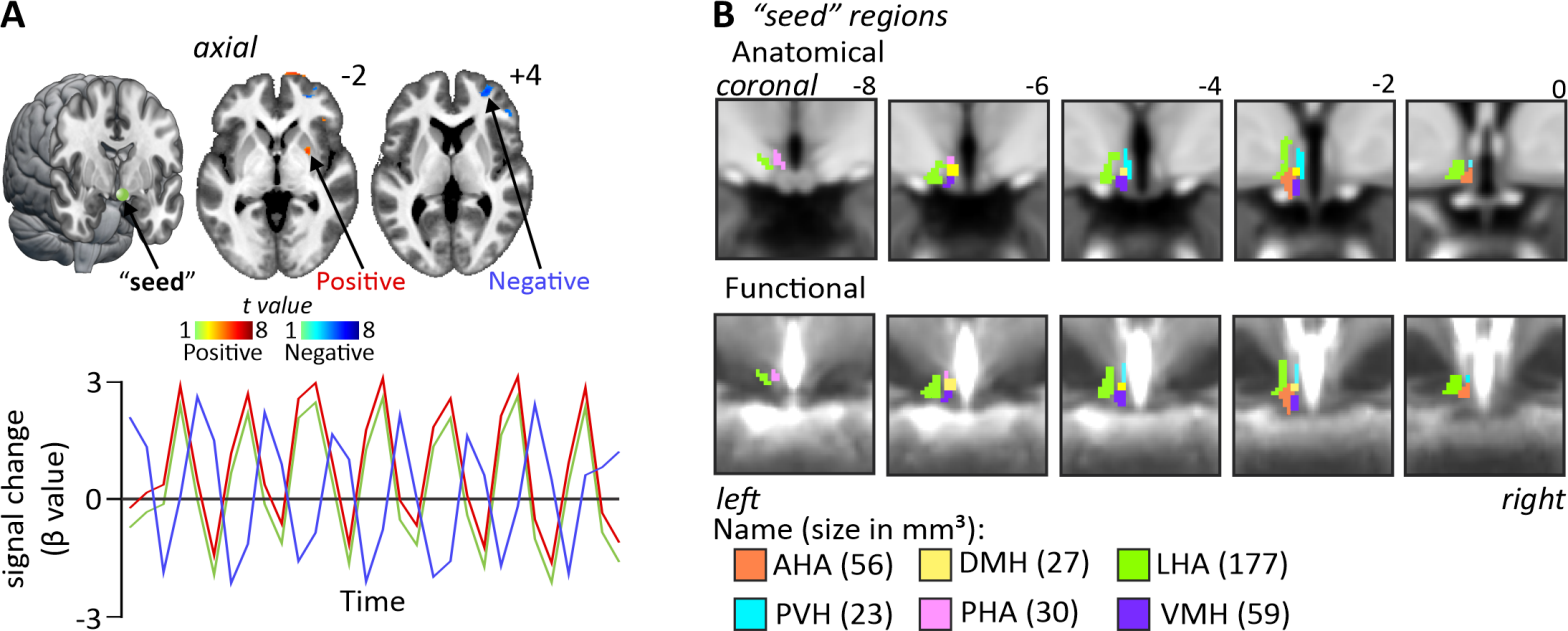
(A) Seed-based resting-state connectivity assesses the covariation strength between the signal extracted from a “seed” region (green sphere) and all other voxels in the brain. Signal covariations can be either correlated (hot colour bar) or anti-correlated (cool colour bar). (B) The six hypothalamic “seed” regions used for the connectivity analysis. The locations and sizes of each of the seed are shown overlaid onto a series of coronal mean T1-weighted anatomical slices (upper row), and mean functional magnetic resonance imaging slices (lower row). Slice locations are indicated to the top right of each image in Montreal Neurological Institute space. For abbreviations, see table.

We recruited 164 healthy individuals, though data from 14 participants were subsequently removed due to excessive movement (see below). The remaining 150 participants were used for the final analyses (83 females; mean[±SEM] age 30.82 ± 0.95 years, range 19–73 years; seeSupplementary Figure 1for age distribution plot). Informed written consent was obtained for all procedures, which were conducted with the approval of the local Institutional Human Research Ethics Committees (ethics reference number: 2019/036), and which were consistent with the Declaration of Helsinki, apart from registration in a public database. All experiments were conducted at the Melbourne Brain Centre Imaging Unit, University of Melbourne.

### 2.2 MRI scanning & data acquisition

Participants lay supine, in a Siemens MAGNETOM 7-Tesla MRI system (Siemens Healthcare, Erlangen, Germany) with a combined tight-fitting single-channel transmit, and 32-channel receive, head coil (Nova Medical, Wilmington MA, USA). A T1-weighted anatomical image set covering the whole brain was collected (MP2RAGE; inversion times TI1 700 ms, TI2 2700 ms; flip angle 1: 4°, flip angle 2: 5°; repetition time 5000 ms; echo time 2.04 ms; image matrix: 224 x224mm; raw voxel size = 0.73 x 0.73 x 0.73 mm; 224 sagittal slices; scan time 7 min). Following this anatomical image acquisition, a resting-state fMRI scan covering consisting of 124 gradient-echo echo-planar fMRI volumes using blood oxygen-dependent (BOLD) contrast and covering the entire brain was collected over a period of 335 s (repetition time 2500 ms; echo time 26 ms; image matrix: 224 x 224 mm; voxel size: 1.0 x 1.0 x 1.2 mm thick; 134 axial slices; bandwidth: 1488Hz/Px; phase partial fourier 6/8; CAIPI factor [slice acceleration] of 4 and a GRAPPA factor [inplane acceleration factor] of 3 with 48 ref lines). Participants were instructed to keep their eyes open and watch a crosshair that was provided on the screen. Images were acquired in an interleaved collection pattern with a multi-band factor of four, an acceleration factor of three, and blipped-controlled aliasing in parallel imaging.

### 2.3 MRI image processing

Statistical Parametric Mapping v12 (SPM12; Wellcome Trust Centre for Neuroimaging, UK) and custom software were used for image analysis. The first 5 brain volumes of each fMRI image series were removed to allow for scanner equilibration leaving 129 fMRI volumes. The fMRI image sets were slice-time corrected, motion corrected, and the 6 directional movement parameters inspected to ensure there was no greater than 1 mm of linear movement or 0.5 degrees of rotation movement in any direction. As mentioned above, fMRI image sets from 14 participants were removed from the final analysis due to excessive movement. The remaining 150 image sets were linear model of global signal detrended to remove global signal changes, physiological noise (relating to cardiac frequency band 60–120 beats/min +1 harmonic), and filtered to remove respiratory (frequency band 8–25 beats/min +1 harmonic) noise using the Dynamic Retrospective Filtering toolbox (Särkkä et al., 2012), and the 6-parameter movement-related signal changes were modelled and removed using a linear modelling of realignment parameters procedure (Macey et al., 2004). The fMRI images were then co-registered to their own T1-weighted anatomical image set, underwent distortion correction using the SynBOLD DisCO toolbox (Yu et al., 2023), and were spatially normalised into Montreal Neurological Institute (MNI) space. Images were resliced into 1 mm isotropic voxels, and isotropic Gaussian filter kernels at full width at half maximum of 2 mm for subcortical structures and 5 mm for cortical structures were used. The smaller smoothing kernel (2 mm) was used for subcortical regions to maintain spatial resolution.

### 2.4 fMRI statistical analysis

**Fig. 2.**
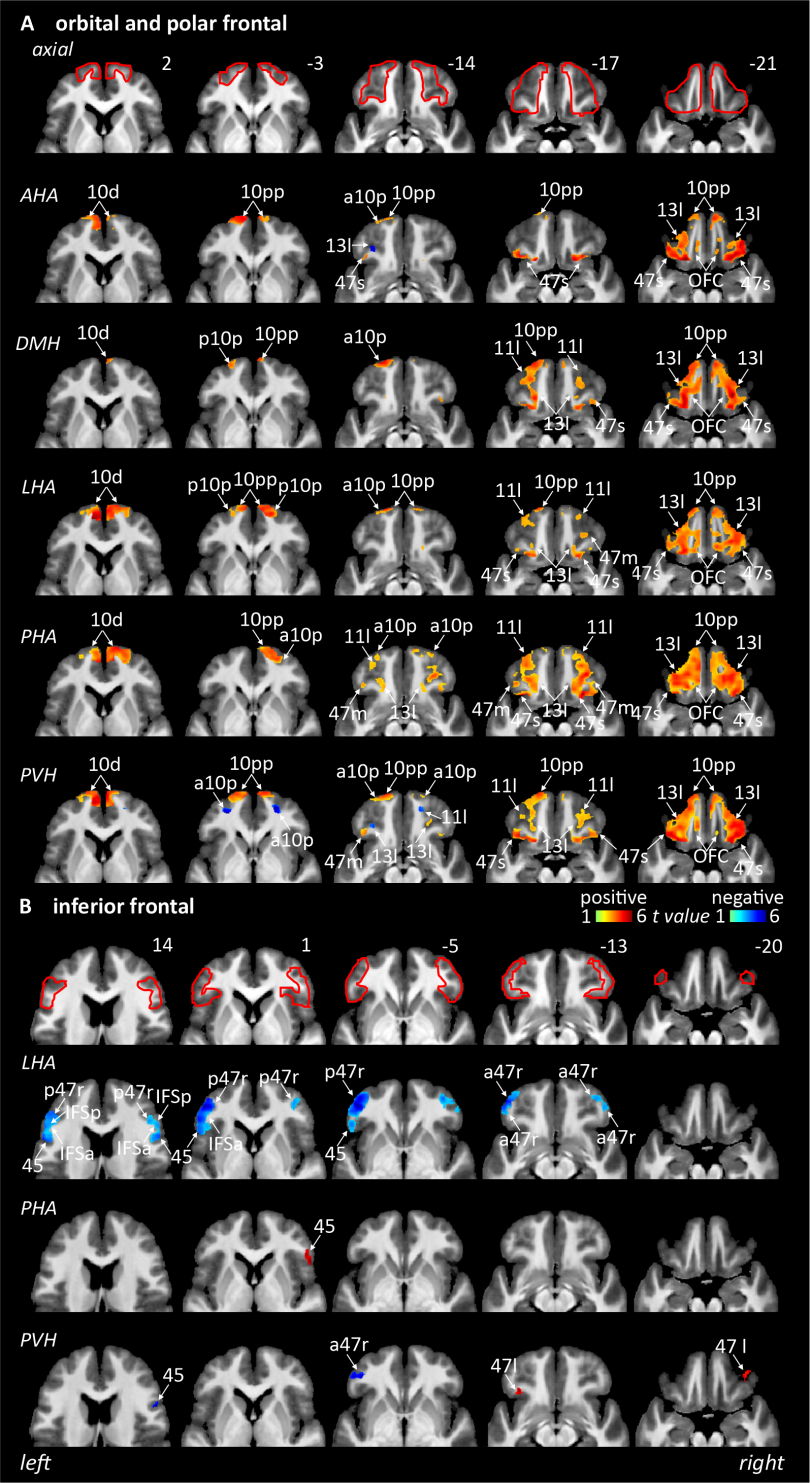
Resting connectivity between six hypothalamic seed regions and the orbital/polar frontal and inferior frontal cortical regions (n = 150). Areas of significant positive (hot colour scale) and negative (cool colour scale) connectivity overlaid onto a series of axial mean T1-weighted anatomical images. Slice locations in Montreal Neurological Institute Space are indicated at the top right of each slice. Red outlines indicating region of interest masked applied for the analysis. (A) Orbital and polar frontal region. (B) Inferior frontal region. For abbreviations, see table.

A*seed*-based connectivity analysis was used to explore signal intensity changes covarying with our seed regions (Fig. 1A). We used a subset of*left*hypothalamic regions (i.e., anterior hypothalamic area, dorsomedial, lateral hypothalamic area, paraventricular, posterior hypothalamic area, and ventromedial), based on (Neudorfer et al., 2020) atlas with slight adjustments so that each*seed*was contained within a grey matter mask made from the mean of all 150 participants fMRI image sets (Fig. 1B). An example of consistency between anatomical and functional images in an individual is shown inSupplementary Figure 2.

To ensure adequate signal-to-noise, we calculated temporal signal-to-noise (SNR) for each of the 6 hypothalamic seeds and found that the mean SNR was suitable, being well above 10 for all seeds (mean [min-max]: AHA: 22.0 [8.5-49.4]; DMH: 29.7 [5.5-50.7]; LHA: 18.3 [5.8-32.0]; PVH: 34.0 [11.2-59.2]; PHA: 26.2 [10.8-46.6]; VMH: 22.3 [8.8-52.0]). Of the 900 SNR ratios calculated (6 seeds x 150 participants), only 15 (0.02%) were below 10 (AHA: 4; DMH: 2; LHA: 5; PVH: 0; PHA: 0; VMH: 4). Furthermore, for all hypothalamus seeds, there were no significant relationships between SNR and age (AHA: r^2^= 0.003 p = 0.54; DMH: r^2^= 0.0003 p = 0.95; LHA: r^2^= 0.01 p = 0.18; PVH: r^2^= 0.009 p = 0.25; PHA: r^2^= 0.001 p = 0.72; VMH: r^2^= 0.001 p = 0.71). We also assessed the potential effects of seed volume on SNR and found no significant relationship; indeed, the seed with the largest volume (LHA) had the lowest SNR (r^2^= -0.6, p = 0.07;Supplementary Fig. 3). Furthermore, we assessed the potential for ‘slice-leakage’ effects where signal from one slice is artificially manifested in another simultaneously excited slice (Todd et al., 2016). On one axial slice, we created two regions of interest (ROI), 10 voxels encompassing the AHA and another 54 encompassing the entire hypothalamus on raw (unprocessed) fMRI images in 10 participants. We then moved these ROIs in the z direction onto 3 aliased and 3 non-aliased slices. We calculated correlation coefficients for signal intensity fluctuations between each AHA and hypothalamus and aliased and non-aliased ROIs, transformed the rho values into z values using Fisher’s z-transformation and z scores, and averaged these values for the aliased and non-aliased ROIs for each participant. Significant differences between aliased and non-aliased ROIs across all individuals were then determined using paired t-tests (p<0.05). We found no significant difference between aliased and non-aliased correlation values (mean±SEM z-value AHA slice aliased: 0.20 ± 0.04, non-aliased 0.31 ± 0.06, p = 0.09; z-value hypothalamus slice aliased: 0.32 ± 0.04, non-aliased 0.36 ± 0.7, p = 0.55) and thus are confident that any ‘slice-leakage’ effects are not significantly affecting our results.

**Fig. 3.**
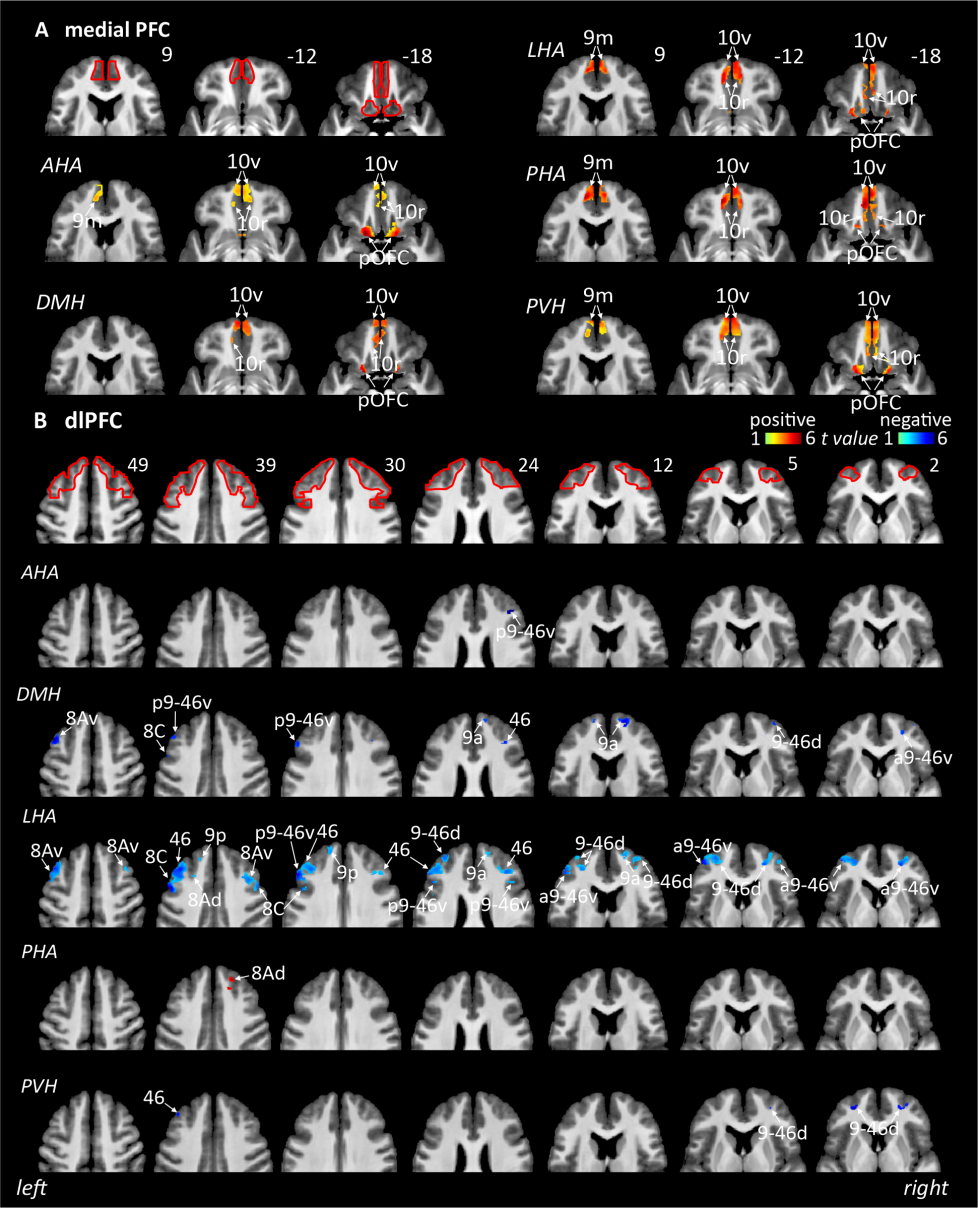
Resting connectivity between six hypothalamic seed regions and the medial prefrontal and dorsolateral prefrontal cortical regions (n = 150). Areas of significant positive (hot colour scale) and negative (cool colour scale) connectivity overlaid onto a series of axial mean T1-weighted anatomical images. Slice locations in Montreal Neurological Institute Space are indicated at the top right of each slice. Red outlines indicating region of interest masked applied for the analysis. (A) Medial prefrontal cortex (PFC). (B) dorsolateral PFC (dlPFC). For abbreviations, see table.

**Fig. 4.**
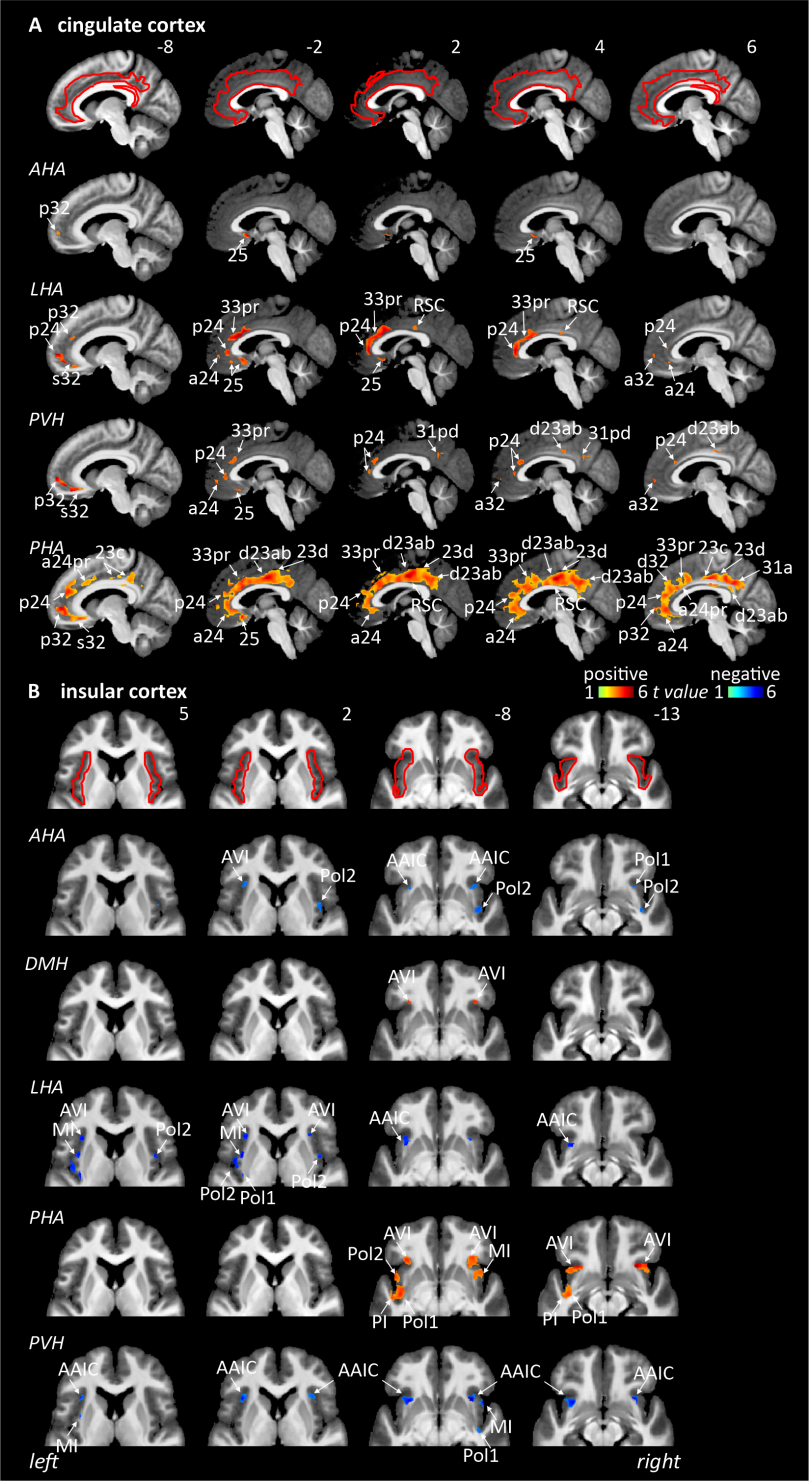
Resting connectivity between six hypothalamic seed regions and the cingulate and insular cortices (n = 150). Areas of significant positive (hot colour scale) and negative (cool colour scale) connectivity overlaid onto a series of sagittal and axial mean T1-weighted anatomical images. Slice locations in Montreal Neurological Institute Space are indicated at the top right of each slice. Red outlines indicating region of interest masked applied for the analysis. (A) Cingulate cortex. (B) Insula. For abbreviations, see table.

**Fig. 5.**
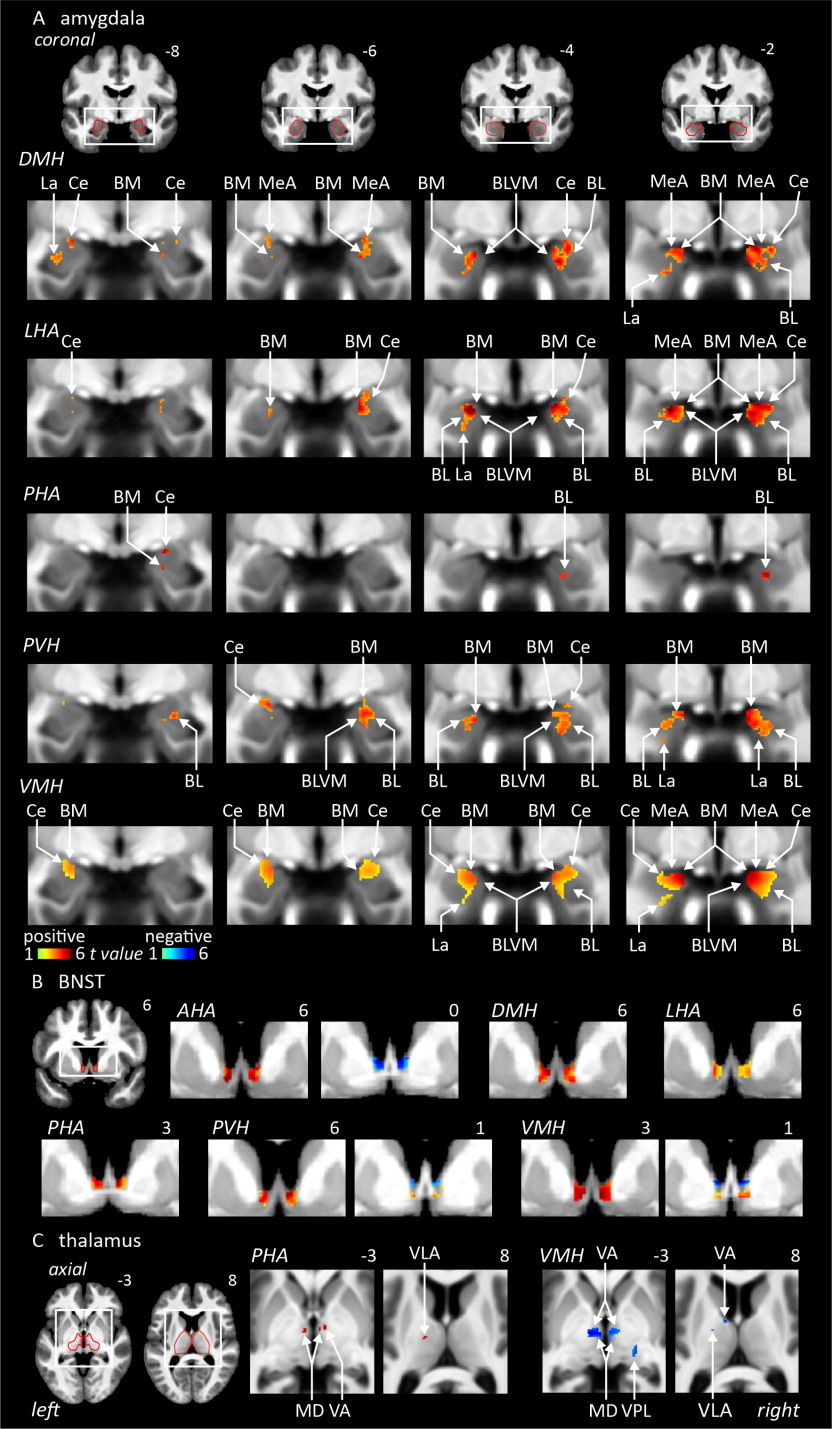
Resting connectivity between six hypothalamic seed regions and the amygdala, bed nucleus of the stria terminalis, and thalamus (n = 150). Areas of significant positive (hot colour scale) and negative (cool colour scale) connectivity overlaid onto a series of coronal and axial mean T1-weighted anatomical images. Slice locations in Montreal Neurological Institute Space are indicated at the top right of each slice. Red outlines indicating region of interest masked applied for the analysis. (A) Amygdala. (B) Bed nucleus of the stria terminalis. (C) Thalamus. For abbreviations, see table.

For each participant, spatial maps of correlation coefficients of each voxel’s time series with that of each of the six seed regions were produced. Since we aimed to explore hypothalamic-limbic system connectivity, we restricted the cortical analysis to the bilateral PFC which was divided in five regions (dorsolateral, medial, inferior, orbital and polar frontal, cingulate) and the insula, and we restricted the subcortical analysis to the bilateral thalamus, amygdala, and bed nucleus stria terminalis (BNST). These cortical and subcortical regions were created using the Human Connectome Project Extended for all regions (Figs. 2-5;Huang et al., 2022). The six correlation coefficient maps for each participant were entered into second-level, one-sample, random-effects analyses to determine significant connectivity increases and decreases for each hypothalamic seed (p < 0.05, false discovery rate corrected for multiple comparisons, minimum cluster size 5 contiguous voxels for subcortical regions and 50 contiguous for cortical regions). Significant clusters were overlaid onto a mean T1-weighted anatomical image for visualisation purposes and included into tables for reference. For cluster location identification, we used the atlas byHuang et al. (2022)in addition toMai and Majtanik (2017)for the amygdala.

To assess whether there were any potentially effects of CSF, we extracted connectivity values from a 3 mm radius sphere placed in the lateral ventricle for each of the 6 hypothalamus connectivity maps in each participant. We found no significant correlations between connectivity strengths in each of the 6 hypothalamus seeds and CSF (AHA: r^2^< 0.001, p = 0.76; DMH: r^2^= 0.002, p = 0.60; LHA: r^2^= 0.003, p = 0.53; PVH: r^2^< 0.001, p = 0.95; PHA: r^2^= 0.007 p = 0.31; VMH: r^2^= 0.001 p = 0.68). In addition, to ensure adequate SNR in each target brain region, we extracted SNR from 9 target regions and found that all had mean SNRs above 20 and in no region was SNR below 10 for any participant (SNR mean [min-max]: BNST: 32.16 [13.74-48.14]; thalamus: 29.87 [16.21-40.74]; amygdala: 30.07 [17.26-42.00]; dlPFC: 59.30 [29.67-78.99]; Inferior frontal cortex: 46.20 [24.77-61.55]; mPFC: 41.90 [21.23-57.74]; orbital polar frontal cortex: 33.27 [17.18-51.03]; insula: 43.25 [24.35-55.58]; cingulate cortex: 47.20 [27.54-61.71]).

## 3 Results

### 3.1 Hypothalamus seed connectivity with the PFC

Analysis of hypothalamic seed connectivity revealed significant connectivity between all hypothalamic clusters and the PFC except for the ventromedial hypothalamus (VMH).

#### 3.1.1 Orbital and polar frontal

**Fig. 6.**
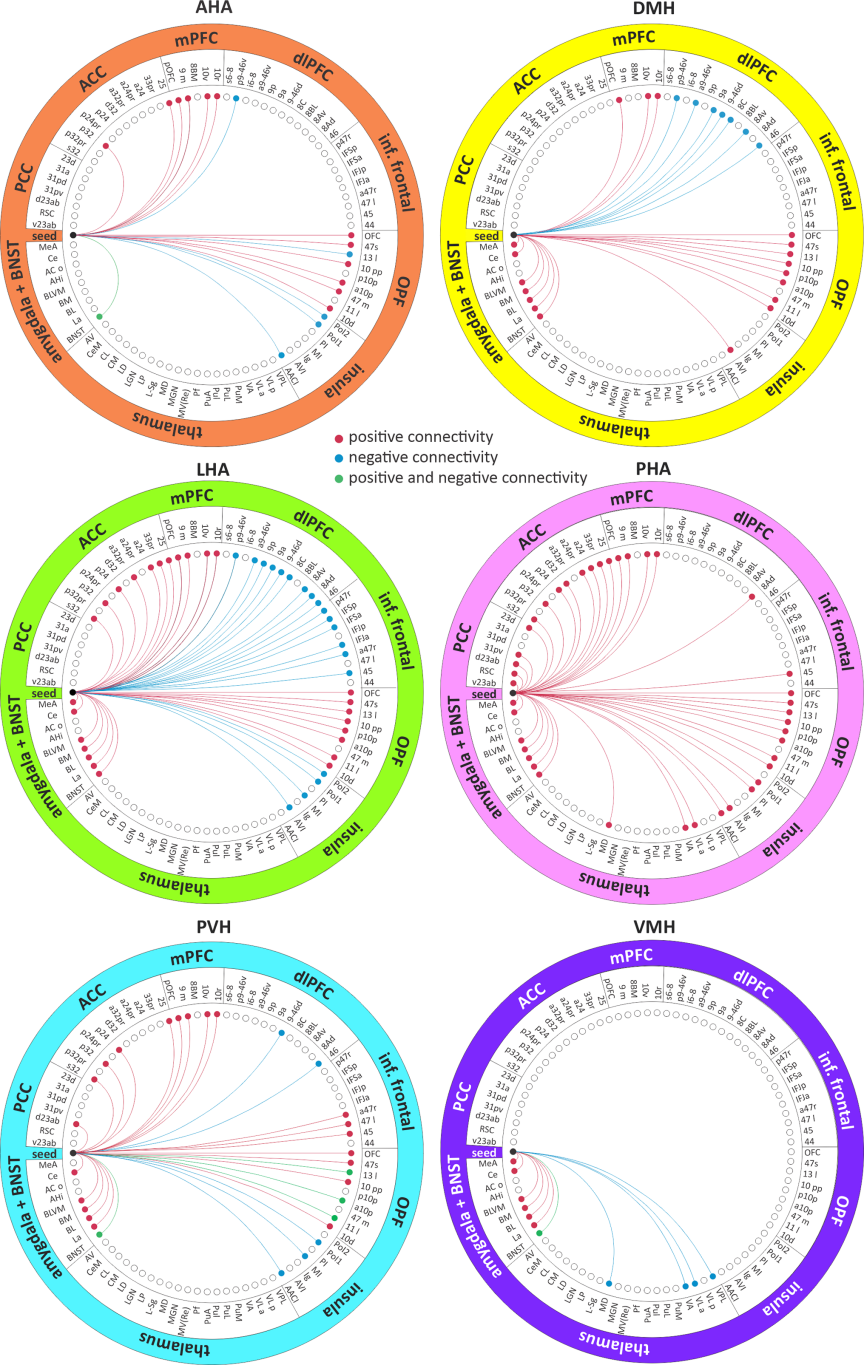
Summary of the six hypothalamic seed resting connectivity patterns. Connectivity between each hypothalamic seed and target region of interest masked on the outer circle and specific subregions in the inner circle. Direction of connectivity described by the colour of the line used from seed to target area. ACC, anterior cingulate cortex; BNST, bed nucleus of the stria terminalis; dl, dorsolateral prefrontal cortex; inf., inferior; OPF, orbital and polar frontal; PCC, posterior cingulate cortex. For abbreviations, see table.

Comprising Brodmann Areas (BAs) 10d, 10 pp, 11 l, 13 l, 47 m, 47s, anterior 10p (a10p), orbital frontal complex (OFC), and posterior 10p (p10p). Similar connectivity to the OPF is observed to be exclusively positive from the dorsomedial (DMH; 47s, 13 l, 11 l, 10 pp, 10d, a10p, p10p, and OFC), lateral hypothalamic area (LHA; 47s, 13 l, 11 l, 10d, 10 pp, a10p, p10p, and OFC), and posterior hypothalamic area (PHA; 47s, 13 l, 11 l, 10 pp, a10p, 10d, p10p, and OFC) while the paraventricular hypothalamus (PVH;*positive*: 47s, 13 l, 11 l, 10pp, a10p, 10d, and OFC;*negative*: 13 l, a10p, 11 l) and anterior hypothalamic area (AHA;*positive*: 47s, 47 m, 13 l, 10pp, a10p, 10p, 10d, and OFC;*negative*: 13 l) are showing more variety (Table 1,Figs. 2A&6).

**Table 1.** Locations, cluster sizes (number of voxels), and t-values for regions that display significant increased or decreased resting-state functional connectivity strengths in cortical regions.

			MNI coordinates				
Seed	Target region		x	y	z	t value	Cluster size
AHA	dlPFC	*Negative*					
		Right p9-45v	40	33	25	4.50	134
	Cingulate cortex	*Positive*					
		Left 25	-4	10	-8	8.31	126
		Right 25	1	10	-8	7.85	126
		Left p32	-8	55	-1	3.81	58
	Orbital & polar frontal	*Positive*					
		Left 47s extending to 47 m & 13 l	-20	16	-23	6.16	2114
		Right 47s extending to 47 m & 13 l	27	18	-23	5.30	2357
		Left a10p extending to 10pp	-19	65	-7	5.22	2710
		Right 10pp extending to a10p & 10d	19	63	-8	4.41	800
		Right OFC	10	23	-23	3.63	90
		Left OFC	-8	31	-21	3.08	173
		Left OFC	-13	48	-23	3.04	67
		Right 10d	11	54	4	2.72	81
		*negative*					
		Left 13 l	-30	33	-14	4.57	88
	Medial PFC	*Positive*					
		Left pOFC	-16	8	-18	8.36	1268
		Right pOFC	17	9	-17	6.95	1152
		Left 10r extending to 10v	-4	63	-2	5.07	1875
		Right 10v extending to 10r	1	48	-22	4.91	1951
		Left 9 m	-9	48	8	3.72	562
		Right 9 m	11	52	4	3.19	94
		Right 9 m	5	56	32	2.80	63
DMH	dlPFC	*Negative*					
		Right 9a	19	55	12	4.59	869
		Left 8Av	-41	14	48	4.58	561
		Right 46	35	30	24	4.23	102
		Left p9-46v	-42	25	37	3.89	324
		Left 9a	-15	58	8	3.69	141
		Right p9-46v	49	32	19	3.64	62
		Right 9-46d	36	57	5	3.60	57
		Left 8C	-47	5	44	3.55	74
		Right a9-46v	26	47	2	3.36	64
	Orbital & polar frontal	*Positive*					
		Right OFC extending to 47s, 11 l, 13 l	25	27	-21	5.72	3353
		Left OFC extending to 47s, 11 l, 13 l, 10pp, a10p, & p10p	-22	24	-21	5.17	5405
	Medial PFC	*Positive*					
		Left pOFC	-17	6	-18	5.37	580
		Right 10v	2	47	-23	5.19	1602
		Left 10v	-1	46	-23	5.08	1399
		Right pOFC	21	7	-19	4.98	363
		Left 10r	-11	48	-9	4.28	102
	Insula	*Positive*					
		Right AVI	34	27	-11	4.89	111
		Left AVI	-30	26	-10	4.24	65
LHA	dlPFC	*Negative*					
		Left 8C extending to 8Av, 46, a9-46v	-46	6	44	5.67	10996
		Right 8Av extending to 8C & 46	35	13	43	4.48	2335
		Right 9-46d extending to a9-26v	25	46	3	4.48	945
		Left 9p	-13	48	32	3.48	308
		Left 9-46d	-15	53	14	3.22	139
		Left 9p	-13	38	41	3.19	107
		Right 8Av	28	2	57	3.14	200
		Right 9a	20	58	13	3.12	328
		Left 8Ad	-19	14	45	2.78	179
		Right p9-46v	40	21	24	2.69	52
		Right 9a	16	51	24	2.69	99
	Cingulate cortex	*Positive*					
		Left p32 extending to s32 & 25	-9	54	1	5.22	885
		Left 33pr extending to a24	-2	22	21	5.06	2220
		Right p32	9	58	-1	4.26	113
		Left p24	-1	34	0	4.23	170
		Right a24	8	35	-10	3.88	96
		Left 25	-2	18	-11	3.88	138
		Right 25	1	15	-10	3.62	68
		Right a32pr	10	33	23	3.25	147
		Right RSC	3	-24	29	3.03	153
	Inferior frontal	*Negative*					
		Left p47r extending to a47r, IFSa, IFSp, 45.	-42	43	-3	6.22	10774
		Right 45 extending to IFSa, IFSp, p47r.	54	12	12	4.04	1801
		Right a47r	38	50	-4	3.61	1694
		Right IFJa	39	23	24	2.76	239
	Orbital & polar frontal	*Positive*					
		Left 10d extending to p10p, 10pp, a10p, 11 l, 47s, 13 l, & OFC.	-8	56	4	6.10	6079
		Right 10d extending to 10pp, p10p, & a10p.	4	60	5	5.02	3062
		Right 13 l extending to 10pp, 47s, & OFC.	23	27	-23	4.80	3889
	Medial PFC	*Positive*					
		Left 9 m	-10	52	2	5.03	552
		Left 10r extending to 10v	-4	64	-4	4.92	1833
		Left pOFC	-20	7	-19	4.68	524
		Right 10v	8	60	-2	4.65	2940
		Right pOFC extending to 10v	20	9	-17	4.25	268
		Right 9 m	5	64	12	4.23	747
		Left pOFC	-2	11	-14	3.34	270
	Insula	*Negative*					
		Left AVI	-30	11	-10	4.36	401
		Right AVI	31	21	-4	4.11	172
		Left MI	-34	2	3	3.71	141
		Left pol1	-32	-19	4	3.55	79
		Left pol2	-41	-3	1	3.40	416
		Right pol2	41	1	4	3.29	68
PHA	dlPFC	*Positive*					
		Right 8Ad	22	32	43	4.35	314
	Cingulate cortex	*Positive*					
		Right RSC extending to d23ab, 23d, 33pr, p24, a24, 25, 31a, p32, d32, s32, a24pr left RSC, 23d, 23c, d23ab, 33pr, p24, a24, a24pr, p32, d32	3	-16	37	5.59	27547
		Left 31pv	-12	-52	29	2.81	128
	Inferior frontal	*Positive*					
		Right 45	54	21	1	4.35	294
	Orbital & polar frontal	*Positive*					
		Right 47s extending to 47 m, OFC, 13 l, 11 l, 10pp, a10p, & 10d.	28	16	-16	6.31	10956
		Left OFC extending to 13 l, 47 m, 11 l, 47s, 10pp, & a10p.	-28	15	-19	5.88	6360
		Left 10d	-3	53	3	4.74	980
		Left 10d	-12	67	13	3.09	97
		Left p10p	-20	58	4	2.31	71
	Medial PFC	*Positive*					
		Left 9 m	-10	51	3	4.96	1271
		Left 10v extending to 10r	-6	41	-19	4.79	2472
		Right 9 m	11	52	2	4.72	1220
		Right 10r extending to 10v	7	56	-7	4.32	2682
		Right pOFC	26	9	-22	4.25	79
		Left pOFC	-24	7	-23	4.05	136
		Right pOFC	17	20	-19	3.46	51
		Right d32	7	38	38	3.36	272
		Left pOFC	-12	20	20	3.30	125
		Left 9 m	-2	44	44	3.01	307
	Insula	*Positive*					
		Left AAIC extending to AVI.	-30	15	-16	5.66	1200
		Right AAIC extending to AVI.	29	17	-13	5.37	1347
		Left PI & Pol1	-38	-10	-11	4.15	985
PVH	dlPFC	*Negative*					
		Right 9-46d	23	49	-2	5.30	112
		Left 9-46d	-25	46	0	5.28	148
		Left 46	-37	30	37	3.91	64
		Left 46	-38	17	42	3.87	53
	Cingulate cortex	*Positive*					
		Left p32 extending to s32 & 25.	-6	56	1	5.82	1067
		Right p32	8	57	-4	5.43	220
		Right p24	4	35	6	3.90	86
		Right a32	5	26	20	3.80	584
		Right d23ab	6	-24	36	3.77	108
		Left p24	-2	38	0	3.71	98
		Right d23ab	3	-54	28	3.36	107
	Inferior frontal	*Positive*					
		Left 47 l	-33	23	-15	5.03	61
		Right 47 l	34	39	-20	4.07	55
		*negative*					
		Right 45	56	11	13	5.33	220
		Left a47r	-40	39	-4	4.42	353
	Orbital & polar frontal	*Positive*					
		Left 10d extending to 10pp, a10p, 11 l, 13 l, 47s, & OFC	-3	62	0	7.09	8416
		Right 47s extending to 13 l, 10pp, OFC, 11 l, a10p, & 10d.	26	13	-23	6.46	6366
		Right OFC	10	22	-23	2.95	54
		*Negative*					
		Right a10p	23	51	-3	5.83	317
		Left a10p	-23	47	-3	5.70	206
		Right 11 l	20	47	-13	4.20	134
		Left 13 l	-29	32	-12	4.19	81
	Medial PFC	*Positive*					
		Left 10r extending to 10v	-3	61	-3	7.66	3027
		Left pOFC	-13	5	-16	6.63	904
		Right pOFC	24	13	-24	6.34	750
		Right 10v extending to 10r	2	47	-22	6.19	2999
		Right 9 m	5	64	12	4.94	1839
		Left 9 m	-3	63	9	4.75	1268
	Insula	*Negative*					
		Right AAIC	32	15	-9	5.56	438
		Left AAIC	-29	13	-10	5.02	1127
		Right MI	44	13	-8	4.22	60
		Left MI	-32	0	6	3.86	53
		Right Pol1	39	-16	-6	3.43	97

Cluster locations are shown in Montreal Neurological Institute (MNI) space.

#### 3.1.2 Inferior frontal

Comprising BAs 44, 45, 47 lateral (47 l), anterior 47r (a47r), IFJa, IFKp, IFSa, IFSp, and posterior 47r (p47r). Fewer regions show connectivity with the inferior frontal area, with the majority from the LHA (*negative*: a47r, p47r, 45, IFSa, IFSp, and IFJa), followed by PVH (*positive*: 47 l;*negative:*a47r, 45) and minor connectivity from the PHA (*positive*: 45) (Table 1,Figs. 2B&6).

#### 3.1.3 Medial PFC

Comprising BA 10r, 10v, 8BM, 9 middle (9 m), and posterior OFC (pOFC). The medial PFC shows consistently positive connectivity from all seeds, the AHA (pOFC, 10r, 10v, and 9 m), DMH (pOFC, 10r, and 10v), LHA (pOFC, 10r, 10v, and 9 m), PHA (pOFC, 10r, 10v, and 9 m), and PVH (pOFC, 10r, 10v, and 9 m) (Table 1,Figs. 3A&6).

#### 3.1.4 Dorsolateral prefrontal cortex (dlPFC)

Comprising BA 46, 8Ad, 8Av, 8B lateral, 8C, 9-46d, 9 anterior (9a), 9 posterior (9p), anterior 9-46v (a9-46v), inferior 6-8 transitional, posterior 9-46v, superior 6-8, and superior frontal language area. While there was very restricted connectivity with the dlPFC for the AHA (*negative:*p9-46v), PHA (*positive correlation*with BA 46), and PVH (*negative*: 46, 9-46d), there was more extensive connectivity with the DMH (*negative:*8Av, 8C, 9a, p9-46d, p9-46v, a9-46v) and even more with the LHA (*negative:*8Ad, 8Av, 8C, 9a, 9p, p9-46d, p9-46v, a9-46v) (Table 1,Figs. 3B&6).

#### 3.1.5 Cingulate cortex

Comprising BAs 33 prime (33pr), a24, 24 prime (24pr), anterior 32 prime (a32pr), dorsal 32 (d32), posterior 24 (p24), posterior 24 prime (p24pr), p32, p32 prime (p32pr), s32, and retrosplenial cortex (RSC). Connectivity with the cingulate cortex followed a similar pattern to that of dlPFC with the major difference of the cingulate showing exclusively positive correlation while the dlPFC was almost exclusively negative, that is, anti-correlated. The most extensive connectivity to the cingulate cortex came from the PHA (v23ab, RSC, d23ab, 31pv, 23d, s32, p32, p24, d32, a24pr, a24, 33pr, 25) and LHA (a32, p32, p24, a32pr, a24, 33pr, & 25) and very extensive connectivity with the PHA (23c, 23d, d23a, d23ab, a24, a24pr, p24, 25). The PVH also showed connectivity to several regions (d23ab, s32, p32, p24, & 25) followed by the AHA (25, p32) (Table 1,Figs. 4A&6).

### 3.2 Hypothalamus seed connectivity with the insula

Comprising the anterior agranular insula (AAIC), anterior ventral insular area (AVI), frontal opercular area, insular granular complex, middle insula (MI), para-insula (PI), piriform, and posterior insula 1 and (Pol 1 & 2) area. The LHA (*negative:*AVI, MI, PoI1, and PoI2), PHA (*positive:*AACI, AVI, PI, PoI1), PVH (*negative:*AACI, MI, and PoI1), and AHA (*negative*: AACI, PoI1, and PoI2) all show similar connectivity patterns with the insula, with the DMH (*positive*: AVI) having more restricted connectivity to a single insula sub-region (Table 1,Fig. 4B).

### 3.3 Hypothalamus seed connectivity with subcortical regions

#### 3.3.1 Thalamus

The thalamus is composed of over a dozen subnuclei, including the anteroventral, central, centro-median, latero-dorsal, lateral geniculate, lateral posterior, limitans suprageniculate, medio-dorsolateral, medio-dorsomedial (MD), medial geniculate, reuniens, parafascicular, pulvinar, ventral anterior (VA), ventral lateral anterior (VLA), ventral lateral posterior, and ventral posterolateral (VPL). We observed significant connectivity to the MD and VA thalamic nuclei from the PHA (*positive*) and VMH (*negative*). We also found connectivity to the VLA thalamus from the PHA (*positive*) and VMH (*negative*). Uniquely, the VMH displayed significant connectivity with the VPL thalamus (*negative*) (Table 2,Figs. 5&6).

**Table 2.** Locations, cluster sizes (number of voxels), and t-values for regions that display significant increased or decreased resting-state functional connectivity strengths in subcortical regions.

			MNI coordinates				
Seed	Target region		x	y	z	t value	Cluster size
AHA	BNST	*Positive*					
		Left	-6	6	-8	7.63	66
		Right	8	6	-3	6.57	60
		*Negative*					
		Left	-5	1	0	6.75	55
		Right	6	2	0	5.69	58
DMH	Amygdala	*Positive*					
		Right BM extending to Ce, MeA, BL, and BLVM	17	-2	-17	5.51	343
		Left Ce extending to BM, MeA, La, and BLVM	-17	-4	-20	5.16	202
		Left Ce	-19	-8	-14	4.39	39
		Left La	-25	-8	-21	3.49	33
		Right Ce	26	-9	-14	2.74	6
	BNST	*Positive*					
		Left	-3	5	-4	5.82	89
		Right	8	5	-8	4.97	86
LHA	Amygdala	*Positive*					
		Left BM extending to La, MeA, Ce, BL, and BLVM	-19	-3	-19	7.66	215
		Right BM extending to Ce, MeA, BL, and BLVM	18	-4	-17	6.61	417
		Left Ce	-24	-11	-12	3.47	16
	BNST	*Positive*					
		Left	-6	4	0	7.67	157
		Right	7	4	-2	5.56	147
PHA	Amygdala	*Positive*					
		Right Ce	21	-8	-13	4.73	10
		Right BL	22	-2	-23	4.44	28
		Right BM	20	-8	-21	3.71	5
	BNST	*Positive*					
		Right	5	3	-1	6.66	83
		Left	-5	2	0	6.31	99
	Thalamus	*Positive*					
		Left MD extending to VA	-4	-8	-3	5.41	15
		Right MD extending to VA	6	-8	-4	4.96	27
		Left VLa	-13	-14	8	4.36	7
PVH	Amygdala	*Positive*					
		Right BM extending to BL, BLVM, Ce, and La	14	-2	-21	5.55	466
		Left BM extending to BL	-14	-2	-19	4.54	116
		Left La	-24	1	-31	4.51	64
		Left Ce	-20	-6	-14	4.25	49
	BNST	*Positive*					
		Right	6	6	-4	8.32	102
		Left	-7	5	-8	7.74	94
		*Negative*					
		Left	-4	1	0	5.65	7
		Right	6	1	1	5.21	14
VMH	Amygdala	*Positive*					
		Left BM extending to Ce, MeA, La, and BLVM	-14	-1	-19	8.42	516
		Right BM extending to Ce, MeA, BL, and BLVM	15	-2	-17	8.10	559
	BNST	*Positive*					
		Right	5	5	-7	7.75	113
		Left	-3	5	-6	7.54	98
		*Negative*					
		Left	-5	1	1	5.11	16
		Right	6	1	1	4.91	21
	Thalamus	*Negative*					
		Right VA extending to MD	8	-1	3	4.93	317
		Left MD extending to VA and VLA	-2	-10	-2	4.88	617
		Right VPL	20	-25	1	4.87	394

Cluster locations are shown in Montreal Neurological Institute (MNI) space.

#### 3.3.2 BNST

The BNST displayed significant bilateral connectivity from all hypothalamic seed regions, the AHA (*positive*and*negative*), DMH (*positive*), LHA (*positive*), PHA (*positive*), and PVH (*positive*and*negative*) (Table 2,Figs. 5&6).

#### 3.3.3 Amygdala

Lastly, the amygdala is composed of several subregions which include basolateral (BL), basolateral ventromedial (BLVM), lateral (La), basomedial (BM), central (Ce), medial anterior, and anterior cortical. Our connectivity analysis revealed widespread connectivity from the DMH (*positive*), LHA (*positive*), PHA (*positive*), PVH (*positive*), and VMH (*positive*) to the Ce amygdala. We also found significant connectivity between the DMH (*positive:*BL, and BLVM), LHA (*positive:*BL and BLVM), PHA (*positive:*BL), PVH (*positive:*BL and BLVM), and VMH (*positive:*BLVM, right BL) and the BL amygdala. Further, the DMH (*positive*), LHA (*positive*), PHA (*positive*), PVH (*positive*), and VMH (*positive*) were significantly connected with the BM amygdala and the DMH (*positive*), LHA (*positive*), PVH (*positive*), and VMH (*positive*) with the lateral amygdala nucleus (Table 2,Figs. 5&6).

## 4 Discussion

Our ultra-high field fMRI data show that discrete hypothalamic nuclei have widespread connectivity to the PFC and associated limbic regions. Overall, the LHA displayed the most widespread, primarily positive, connectivity to the PFC, followed closely by the PHA, PVH, DMH, and AHA. In contrast, the VMH displayed no cortical connectivity. Similarly, all hypothalamic seed regions apart from the VMH displayed connectivity with the insula, although the DMH only connected with the anterior ventral insula region. The PHA and DMH displayed positive, while the AHA, LHA, and PVH displayed negative, insula connectivity. All hypothalamic seeds demonstrated significant positive connectivity with the BNST, with the AHA, PVH, and VMH also displaying negative connectivity as well. Although all hypothalamic seeds, except for the AHA, displayed positive connectivity with the amygdala, only the PHA and VMH hypothalamic seeds displayed positive and negative connectivity with the thalamus, respectively (seeFig. 6for summary). These findings largely recapitulate those of non-human primate anatomical tract tracing studies that demonstrated widespread connectivity to cortical regions and consistent links to subcortical areas (Floyd et al., 2001;Öngür et al., 1998). Our data provide a new platform for investigating altered regional hypothalamic function in numerous conditions.

Experimental animal studies described two key PFC networks, an “orbital” and a “medial” network. The “medial” prefrontal network is closely linked to regions critical for emotional processing which comprise the mPFC, ACC, posterior OFC, and anterior insular (*human Brodmann’s areas:*14, 24, 25, 32, 10r, rostral orbital area 10p, 11, and agranular insular area, posterior OFC;*monkey Brodmann’s areas:*BAs 14r, 14c, 24, 25, 32, 10 m, 10o, 11 m), whereas the “orbital” prefrontal network is closely linked to regions essential for sensory processing which comprise the lateral and central orbitofrontal cortex (*including human Brodmann’s areas:*OFC, 11 l, 13, and 47; monkey*Brodmann’s areas*: 13 l, 13 m, 13b, 12 l 12 m, and 12r) (Carmichael & Price, 1996;Öngür et al., 2003). Subsequent neuroanatomical tract tracing studies in animals have revealed that the hypothalamus is more closely connected to the medial prefrontal network, with the posterior, anterior, and dorsal hypothalamic nuclei projecting to and receiving inputs from subregions of this medial network in both monkeys (Freedman et al., 2000;Öngür et al., 1998) and rodents (Floyd et al., 2001). These anatomical connectivity patterns are largely consistent with our functional connectivity results in which we found extensive connectivity between the posterior, anterior, and dorsal hypothalamus with areas of the medial PFC network. In addition, our results reveal connectivity between the posterior, anterior, and dorsomedial hypothalamus and a number of subregions of the orbital PFC network. The LHA was the only region showing consistent connectivity with both medial and orbital networks, which is consistent with monkey and rat tract tracing.

In contrast, while we found PVH connectivity with both the medial and orbital networks, this is in contrast to tract tracing in the money, which did not report connections between the PVH and these cortical areas (Öngür et al., 1998). However, since this previous tract tracing investigation was limited to cortical tracer injection sites, it is difficult to ascertain whether the full extent of cortical projections to the PVH was evaluated. Indeed, rodent tracing studies have revealed PVH connectivity with some subregions of the medial network, such as the caudal and rostral prelimbic cortex. Interestingly, we found no significant VMH functional connectivity with either the medial or orbital networks. This finding is consistent with a rodent study which observed no structural connectivity from the cortex to the VMH (Floyd et al., 2001), although a primate tract tracing study reported structural connectivity between the VMH and the ACC, OFC and agranular insula (Öngür et al., 1998). It might be the case that the lack of resting functional connectivity may be due to sex-related effects or that robust signal covariations occur during evoked tasks such as during acute noxious stimuli. Indeed, we have previously shown that acute noxious stimuli evoke signal intensity changes in the VMH that are sex-specific, that is, only occur in males (Robertson et al., 2022). It might be that painful stimuli drive changes signal covariations between the VMH and cortical regions in males only, or that at rest, sex has a significant effect on VMH-cortical connectivity.

All hypothalamic seeds displayed connectivity with the BNST, and all apart from the AHA with the amygdala. Experimental animal tract-tracing studies have shown that the amygdala and BNST each have reciprocal connections with the DMH, LHA, PHA, PVH, and VMH (Abrahamson & Moore, 2001;Dong & Swanson, 2004a,2004b;Mehler, 1980;Torrisi et al., 2015). While the AHA has been reported to have extensive connections with the BNST (Abrahamson & Moore, 2001;Dong & Swanson, 2004a,2004b), verifying connections between the AHA and amygdala is more challenging. In rodent studies, AHA-amygdala projections have been shown (Conrad & Pfaff, 1976;Ottersen, 1980) and in a non-human primate study, extensive projections from the AHA to most regions of the amygdala are reported (Öngür et al., 1998). However, in the latter study, the authors state the possibility that the medial preoptic hypothalamus and anterior commissure may also have taken up the tracer which, given the presence of extensive connections between these regions and the amygdala (Berk & Finkelstein, 1981;Chiba & Murata, 1985), makes the results difficult to interpret with accuracy. Our data suggest that at least with respect to functional connectivity, AHA-amygdala signals do not covary or are very subtle at rest when neural activity is dominated by ongoing fluctuations in brain circuits such as the default mode network. Interestingly, most of the hypothalamic connectivity with the amygdala was in the more ventral aspects, and it has been previously noted that the medial network connects most strongly with the ventral (basal nucleus) amygdala supporting the idea that the hypothalamus is linked more closely to the medial network (Carmichael & Price, 1996).

Within the BNST, positive hypothalamic connectivity was largely within the anterior region, while negative correlations with the AHA, PVH, and VMH were in the more posterior region. In rodents, an anterior/posterior BNST divide has been reported with the anterior region largely projecting to the PFC, amygdala, and brainstem, while the posterior region is projecting more robustly to the hypothalamus and amygdala (Lebow & Chen, 2016). This division is consistent with our finding of positive hypothalamic connectivity with the anterior BNST and negative hypothalamic connectivity with the posterior BNST. This suggests that differences in the direction of correlation (positive or negative) may reflect functional differences in the anterior and posterior BNST. Finally, the mediodorsal, ventral anterior, and ventral lateral thalamic nuclei displayed extensive negative connectivity with the VMH and positive correlations with the PHA. The mediodorsal thalamus is known to have extensive links to limbic regions such as the PFC and central and basolateral amygdala (Aggleton & Mishkin, 1984;Kuroda et al., 1998) and has been observed to be a key relay centre between these limbic regions (Parnaudeau et al., 2018). Our finding of no significant connectivity between the VMH and the cortex may be explained by the strong VMH-mediodorsal thalamus connectivity which may be relaying VMH signal changes to cortical areas. This is supported by the connectivity between the VMH and other subcortical regions such as the amygdala and BNST, suggesting that the VMH at rest may have a tonic link to subcortical areas. In contrast, the PHA demonstrated strong connectivity with both thalamic and several cortical regions, which may suggest a network of communication between the PHA, thalamus, and regions for the PFC and insula occur at rest.

Our data provide a platform for exploring alterations in connectivity patterns between various hypothalamic regions and the limbic system. Indeed, hypothalamus resting-state connectivity has been shown to be altered in several conditions, including migraine (Ferraro et al., 2018;Meylakh et al., 2020), metabolic disorders (Kullmann et al., 2014,^2023^;Le et al., 2020), and psychiatric conditions (Tu et al., 2018;Wang et al., 2019;Xiao et al., 2024;Zhang et al., 2018). In a number of these studies, attempts were made to distinguish between regional areas of the hypothalamus such as medial, lateral, anterior, and posterior, although none had the spatial acuity to robustly target specific hypothalamic regions as we had done in this investigation (Ferraro et al., 2018;Kullmann et al., 2014;Meylakh et al., 2020;Tu et al., 2018;Zhang et al., 2018). Activation of hypothalamic nuclei has only been observed at 7-Tesla in healthy controls during painful stimulation (Robertson et al., 2022) and placebo paradigm (Crawford et al., 2023). No attempt has been made to observe resting-state connectivity of the hypothalamus at 7-Tesla or at lower resolution in a uniquely healthy population, while incidental reports of healthy participants’ hypothalamic connectivity were not able to capture the resolution of individual nuclei (Kullmann et al., 2014).

There are, however, some limitations to the current study that need discussing. Firstly, resting-state connectivity measures functional signal covariation at infra-slow frequencies and implies that areas that display similar signal fluctuation patterns are ultimately connected anatomically. Of course, this may not be true for all connections. Another MRI technique, diffusion-weighted imaging can be used to detect fibre directionality and with modern analysis methods such as Tract-Based Spatial Statistics can be used on a group level to understand anatomical connectivity of regions within the human brain. With hardware and software improvements, this method could be potentially used to define hypothalamic anatomical tracts as it has already been implemented on large brain regions (Mathiopoulou et al., 2023) as well as discrete brainstem nuclei, including sub-regions of the midbrain periaqueductal grey matter and even a discrete brainstem-diencephalic tract (Ezra et al., 2015;Kaya et al., 2025). Secondly, while we created hypothalamic seeds using established atlas, there is inevitably some inter-individual variation in the precise location of each hypothalamic nuclei. Given the different seed connectivity patterns, we suggest that any small anatomical variations between individuals did not affect the overall robustness of the observed functional connectivity patterns. Thirdly, while it is common for studies to use similar scan times as we did for resting state fMRI (~5 min), our relatively longer repetition time reduced the number of brain volumes typically acquired. Fewer brain volumes can limit the ability to accurately capture signal fluctuations, reduce statistical power, and reduce reliability (Birn et al., 2013). While these are legitimate limitations, we are confident that the large dataset (n = 150) reduced the impact of these potential confines and that our results are robust and reproducible. Fourthly, it has been shown that age can have an effect on resting-state connectivity, with some age-related strength declines in many networks (Varangis et al., 2019). While we found no significant effects of age on SNR in all hypothalamic seeds, it is possible that the connectivity patterns of these seeds alter with age. A future study exploring this potential effect would be valuable. It should be noted that we have explored signal covariations within a region of the brain in which the function of small neighbouring nuclei can vary dramatically. We used a small smoothing kernel in the hypothalamus to limit smoothing signal fluctuations from one nucleus to a neighbouring one. Furthermore, there is the potential that partial volume “bleeding” effects could have resulted in decreased signal “purity” in each hypothalamus seed. However, given that the overall patterns of connectivity varied between hypothalamus seeds, it is likely that the effects of these potential constraints are limited. Finally, we explored only the left hypothalamus as we were interested in defining laterality of any connections and exploring both sides individually would have made the manuscript overly dense and difficult to follow. Indeed, there is some evidence that details laterality effects for areas including the DMH, PVH, and MPO (Fontes et al., 2017;Sullivan & Gratton, 2002;Xavier et al., 2009).

In conclusion, we have shown that regionally the hypothalamus interacts with the PFC, insula, and associated limbic areas in unique manners which may be linked to functional differences. Preclinical rodent and non-human primate findings largely support these findings with some potential key differences. This may guide research into potential differences in the role of the hypothalamus in human pathologies.

## Supplementary Material

Supplementary Material

## Data Availability

Research data are not shared due to human ethics requirements.
