# Supplementary material for: Regional hypothalamic resting state connectivity with limbic structures: An ultra-high field functional magnetic resonance imaging investigation
Source: Imaging Neurosci (Camb). 2025 Jun 16;3:IMAG.a.46. doi: 10.1162/IMAG.a.46 (PMC12319977; doi:10.1162/IMAG.a.46)
Supplement: Supplementary Material [file imag.a.46_supp.pdf]

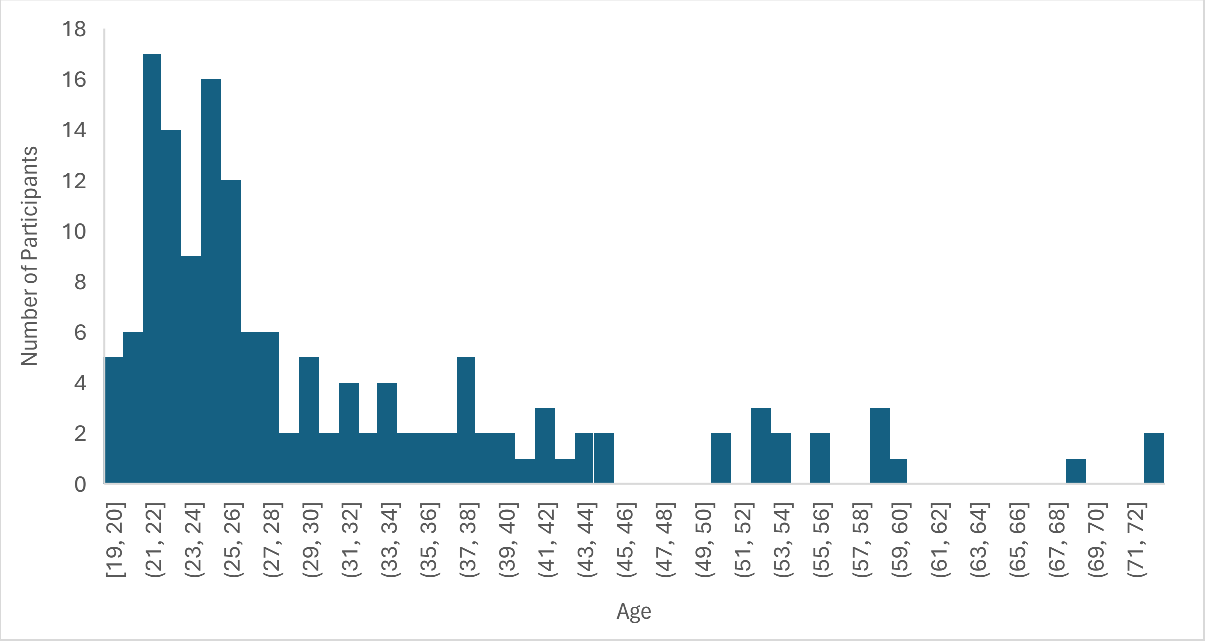

Supplementary Figure 1. Distribution of ages in the current dataset.

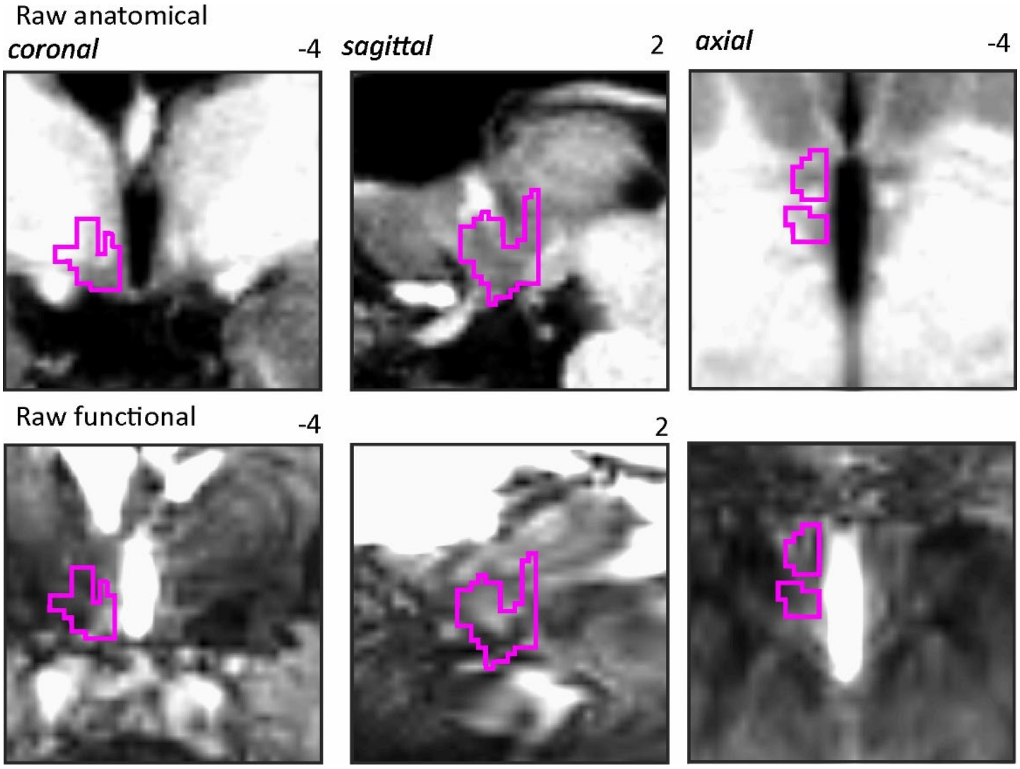

Supplementary Figure 2. The localisation of the hypothalamus using a mask outlined in pink overlaid onto an individual's T1-weighted anatomical slices (upper row), and their functional magnetic resonance imaging slices (lower row). Slice locations are indicated to the top right of each image in Montreal Neurological Institute space.

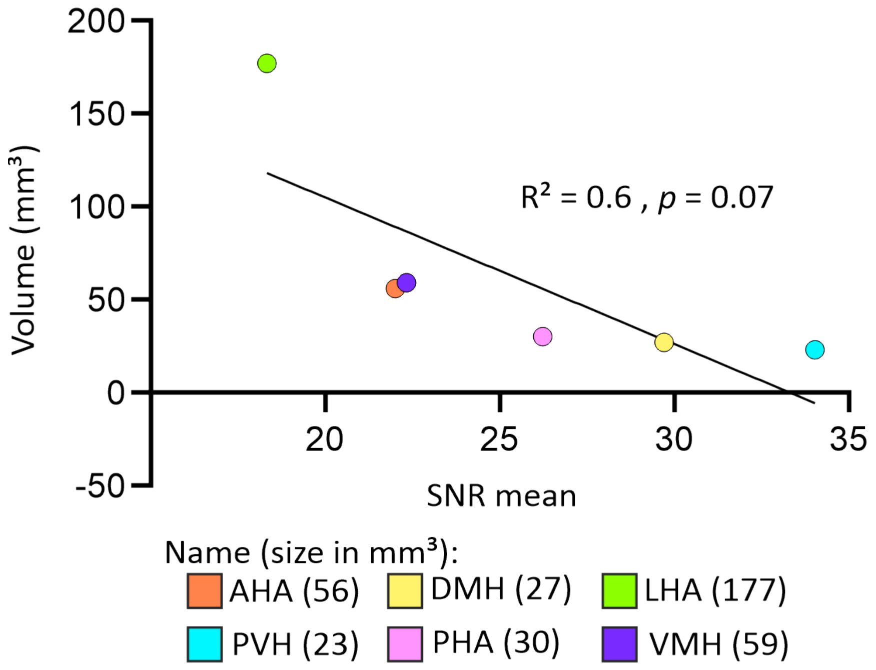

Supplementary Figure 3. Signal-to-noise ratio (SNR) mean plotted for regions of interest of this study against their volumes. For abbreviations, see table.
